# Editorial: Role of Lipids in the Dynamics of Allergic Airway Inflammation

**DOI:** 10.3389/fimmu.2020.612297

**Published:** 2020-11-04

**Authors:** Nestor González Roldán, Katarzyna Anna Duda

**Affiliations:** Group of Allergobiochemistry, Priority Research Area Asthma & Allergy, Research Center Borstel, Leibniz Lung Center, Member of the German Center for Lung Research (DZL), Airway Research Center North (ARCN), Borstel, Germany

**Keywords:** lipid adjuvants, natural killer T cell, asthma, structure-activity relationship, allergic inflammation, allergen-specific immunotherapy

Allergic airway inflammation is a multifactorial and complex process considered to be an abnormally exacerbated reaction towards otherwise innocuous common environmental factors, such as pollen grains, dust mites, or their molecular components. It involves several cell types of the innate and adaptive immune system, including airway epithelial cells, ILC2, Th2 cells, mast cells, eosinophils, and basophils. The Th2-related and prototypical cytokines IL-4, IL-5, IL-9, and IL-13; and the production of allergen-specific IgE are also part of this type of inflammation. When allergic airway inflammation becomes chronic, it leads to the development of asthma. At an early age, allergic sensitization towards proteins takes place in susceptible individuals. In most cases, allergens are proteins from diverse origins and functions, which under normal circumstances would not be recognized by the host’s immune system as harmful or dangerous (antigenic). Thus, over the course of decades, protein-allergens have been the focus of research regarding allergic airway inflammation. But, what makes a protein become an allergen? Despite the significant progress in the identification and molecular characterization of allergens, this key question remains unanswered or can only be partially explained, indicating that there are other factors involved. As highlighted by Platts-Mills (1), allergens do not arrive in the airways alone. Their delivery occurs exclusively in particles, which consist of a complex mixture of chemically different molecules, including lipids. Hence, allergic sensitization is a multifactorial process that is not only regulated by the intrinsic biological activity (i.e. protease activity) of the allergens but also by bioactive lipids. Pollen grains evidence very clearly this concept, as they have a rich lipidome of their own (Dahl). These external lipids are either associated to the allergens as ligands, as a component of the allergenic particles, or are derived from microorganisms present on the allergen source (Lembo-Fazio et al.).

By definition, lipids are hydrophobic or small amphipathic molecules, classified into eight well-defined categories: fatty acids, glycerolipids, glycerophospholipids, sphingolipids, sterol lipids, prenol lipids saccharolipids, and polyketides (2). It has been shown that endogenous lipid mediators are crucial for the control of antigen presentation by dendritic cells in the context of asthma (Debeuf and Lambrecht), and for the reactivity of mast cells during the effector phase of allergic inflammation (Hagemann et al.). There is now a growing body of evidence indicating that not only endogenous, but exogenous lipids (not synthetized by host’s cells) present in allergenic particles, can target different cell populations of the immune system (3) (Lee et al.). Some examples are the phytoprostanes released from grass pollen were shown to prime dendritic cells towards glycolipid presentation and enhance mast cell degranulation (González Roldán et al.). And a glycolipid isolated from *Aspergillus fumigatus* activated NKT cells, inducing airway hyperresponsiveness in an experimental model of asthma (4). Therefore, it is very likely that lipids are the “missing” additional factor involved in the regulation of what we call the “dynamics of allergic inflammation”: the transition from one disease stage to the next one, sensitization-chronification-exacerbation. Following this idea: in susceptible subjects during the early phase of exposure, lipids contained in the allergenic particles (working as an adjuvant) could favor the release of the early IL-4 required for the switch to produce IgE by B-cells, resulting in sensitization (5). Once the individual is sensitized to a particular allergen, the repeated contact with bioactive lipids, could provide a background “pre-activation” status, by influencing cellular processes such as calcium mobilization or by keeping a pro-allergic microenvironment. And finally, once allergic inflammation has become chronic, the encounter with allergenic particles has the potential to provoke an exacerbation on its own, but the additional presence of lipids, either alone or working in a synergistic manner, potentiate the intensity of the response.

A plausible explanation why lipids have remained out of focus of study in the field of allergic inflammation, may reflect the challenges in working with lipids under laboratory conditions. With the exception of lipid mediators, the majority of lipids are water-insoluble. Under hydrophilic conditions lipids tend to form micelles or adhere to the walls of tests tubes, becoming not bioavailable. Thus, their hydrophobic properties hinder the analysis of their biological activity in water-based media cultures used in *in vitro* systems. To overcome this issue, researchers often try to solubilize lipids using organic solvents such as DMSO or ethanol, or detergent solutions, that are either toxic for the cells or do not reflect the natural way of delivery to host’s cells. In nature, lipids are often bound or associated to diverse lipid transfer proteins, allowing their delivery in a hydrophobic milieu to the target cells (Scheurer and Schülke). The lipid-allergen association can protect allergens from degradation and increase their allergenicity by bringing its own lipid-adjuvant, and thus enhancing the sensitization towards these proteins (Jappe et al.).

Speaking of lipid classes, one has to take into consideration the existence of a high structural/chemical heterogeneity between and inside each class. And subtle structural changes like the targeted addition/removal of functional groups can render lipids either inactive, make them more potent or even induce a shift of the inflammatory response (i.e. Th1 *vs.* Th2). This structure-activity relationship can be exemplified looking at the recognition of glycolipids presented on CD1d by NKT cells. The length of the fatty acid chains and the presence, and location of double bonds affects their affinity for the hydrophobic pockets of CD1d required for loading; and influences the orientation of the carbohydrate part that is recognized by the TCR of NKT cells. As a consequence, these structural cues determine the type of cytokine response by NKT cells (6). However, for other lipid classes than glycolipids, the structure-activity relationship behind the inflammatory response towards an allergic phenotype remains poorly understood. We believe that further detailed structural work, along with the elucidation of receptors, pathways, and cells involved in lipid recognition will set the rational base for the use of lipids as adjuvants for the development of suitable allergen-lipid formulations directed to be used as prophylactic for individuals at risk of developing allergy, or for allergen-specific immunotherapy.

With this Research Topic we aim to call readers’ attention towards lipids as important regulators of allergic inflammation, provide an overview of current knowledge, with a focus on structure-activity relationship.

## Author Contributions

NG and KD wrote the manuscript. All authors contributed to the article and approved the submitted version.

## Conflict of Interest

The authors declare that the research was conducted in the absence of any commercial or financial relationships that could be construed as a potential conflict of interest.
